# 4D‐Printed Spin Crossover Metamaterials with Giant Programmable Positive or Negative Thermal Expansion

**DOI:** 10.1002/adma.202522073

**Published:** 2026-04-14

**Authors:** Adelais Trapali, Yuteng Zhang, Seyed Ehsan Alavi, Nagham Mawassy, Raja Zulkarnain, Gábor Molnár, Lionel Salmon, Azzedine Bousseksou

**Affiliations:** ^1^ CNRS & Université de Toulouse (UPS, INP) LCC Toulouse France

**Keywords:** 4D printing, coefficient of thermal expansion, fused deposition modelling, mechanical metamaterials, polymer composites, spin‐crossover

## Abstract

In the past decade, 3D‐printed cellular materials have witnessed an impressive advancement affording a wealth of remarkable mechanical properties, such as negative Poisson's ratio, negative compressibility, and negative coefficient of thermal expansion (CTE). Recent efforts in this field have been increasingly considered 4D‐printed metastructures, which leverage shape‐morphing properties of stimuli‐responsive materials. Here, we introduce a new class of 4D‐printed metamaterials based on bistable spin crossover (SCO) molecular materials. These systems synergistically couple dissimilar materials at different size scales to harness mismatched thermomechanical properties—specifically differential thermal expansion and stiffness—to generate large directional deformations upon heating or cooling. Through a combination of theoretical modeling and experimental validation, we demonstrate that our SCO‐based 4D‐printed structures can achieve programmable motions, including positive and negative expansion. The associated CTE reaches peak values of ca. +14400 and −11400 ppm/°C, respectively, more than 10 times greater than those reported in the literature for 3D‐printed analogues. This work establishes a versatile and generalizable conceptual strategy for engineering multilevel, hierarchical architectures with programmable functionalities, advancing the design of energy‐efficient soft actuators and reconfigurable/adaptive material systems.

## Graphical abstract

Spin crossover composite filaments are implemented for the rational design of 4D‐printed mechanical metamaterials. The obtained bi‐material structures guided by a theoretical analysis based on analytical calculations and Finite Element Analysis simulations exhibit giant programmable positive or negative deformation with coefficients of thermal expansion up to 14000 ppm/°C.

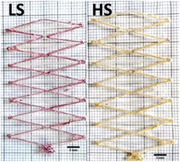

## 1 Introduction

Materials that undergo controllable mechanical transformations under external stimuli form the basis of next‐generation actuators, shape‐morphing devices, and soft robotic systems [1, 2, 3, 4, 5, 6, 7, 8]. Among various actuation mechanisms, thermal response is particularly attractive due to its simplicity, tunability, and compatibility with environmental energy sources [9, 10]. However, conventional materials often lack the ability to convert uniform temperature changes into significant, directional, and programmable motion. Architected metamaterials, in which the geometry governs the mechanical behavior more than composition, offer a promising route to overcome these limitations [11, 12]. By spatially arranging materials with contrasting thermal and mechanical properties, one can engineer internal stress fields that convert thermal input into sophisticated mechanical responses. These responses can be amplified and directed by tuning geometric features such as unit cell shape, orientation, and connectivity. Notably, recent progress in metamaterial design, allowed for significant tunability and amplification of their coefficient of thermal expansion (CTE), demonstrating the potential of these metastructures for a range of applications requiring control of thermal strain, including precision instrumentation, optical, and aerospace systems [13, 14, 15, 16, 17, 18, 19, 20, 21, 22].

To implement these novel programmable CTE metamaterial concepts, 3D printing has emerged as a key enabling technology, allowing swift and accurate reproduction of complex geometries whose properties align with theoretical models. Up to now, however, most efforts have focused on the structural design using conventional 3D printable materials (mostly polymers). It appears thus vital to develop 3D‐printing techniques with advanced functional materials to introduce novel and/or enhanced functionalities, which leverage synergistically cutting‐edge structural and material designs. An important result was the programmable positive and negative CTE up to ca. 700 ppm/°C obtained for an additively manufactured bi‐material metamaterial with diamond cell based on nylon and polyvinyl alcohol (PVA) [18]. In this context, 4D‐printing, which refers to the additive manufacturing of stimuli‐responsive, shape morphing materials [23, 24, 25], emerged as a particularly appealing approach. Among the different “active materials” used for 4D printing, shape memory polymers, liquid crystal elastomers, and hydrogels, in combination with a range of different stimuli (light irradiation, electric/magnetic fields, pH, heat …), have been the most popular [26, 27, 28, 29, 30, 31]. The resulting 4D‐printed metastructures have found applications in soft robots, smart actuators, self‐deployable and energy‐absorbing structures [32, 33, 34, 35, 36].

Here we report a 4D‐printed metamaterial concept affording programmable, colossal CTE (both positive and negative) through a rationally designed, multi‐material architecture. As active, thermo‐responsive materials, we use spin crossover (SCO) molecules, which are well‐known for exhibiting abrupt and reversible changes in volume and stiffness in response to temperature variations, due to electronic transitions between the low‐spin (LS) and high‐spin (HS) molecular states [37, 38, 39, 40, 41]. Our strategy to transform weak, molecular‐scale forces into a large, muscle‐like contraction integrates three design levels. It starts with the chemical design space, since the CTE of bulk SCO materials is dictated by their chemical composition and intrinsic crystal structure. In the present study, we use a benchmark iron‐triazole complex, [Fe^II^(4‐NH_2_‐1,2,4‐triazole)_3_]SO_4_ (**1**), chosen for its outstanding actuating properties in terms of mechanical strain, work density, and stability [42]. Nevertheless, it is important to note that chemical modifications, such as counter‐anion, metal, or ligand doping, can be used to tune the SCO properties [37, 38, 39], for example, to adjust the transition temperature to specific applications [43]. However, neat SCO materials cannot be easily integrated into mechanical systems and their use has been restricted mainly to micro/nano‐scale devices [44]. Hence, at the next stage, particles of **1** are embedded in a polymer matrix. Here, the key design parameters are related to the microstructure of the constituents (morphology, orientation, connectivity, volume fraction, …) and to the thermomechanical properties of the matrix. Our previous investigations of the mechanical properties of various SCO@polymer composites allowed us to maximize their CTE at 4000 ppm/°C for only 25 vol.% SCO concentration [45]. Remarkably, this colossal CTE in the dilute composite was in excess of that of the bulk material. Micromechanical modelling revealed that this type of strain amplification can be readily achieved when preferentially oriented, rod‐shaped particles are embedded into soft matrices, such as thermoplastic polyurethane (TPU) [46]. These SCO composites were then successfully used to fabricate bilayer bending actuators, which showed precise and reliable electro‐thermal actuation with fast, pre‐programmed, directional movements and even self‐healing ability [47, 48, 49]. Importantly, using an appropriate closed‐loop control, these light‐weight actuators could be deployed without any external sensing or heating elements [50].

In the present study, we implement a third design level, wherein SCO composite **1**@TPU building blocks with high CTE are assembled, together with “passive” units, into dual‐material lattice structures. To fabricate these architected materials, we developed SCO‐based composite filaments compatible with FDM (fused deposition modeling) printing. Our combined theoretical and experimental results demonstrate that by tuning material placement and geometry, the selected deformation mode—expansion or contraction—can be amplified to reach colossal values, which can be predicted and programmed. These results pave the way for a class of rationally‐designed 4D‐printed mechanical metamaterials, outperforming analogous 3D‐printed objects, thanks to the integrated shape‐morphing SCO component.

## 2 Results and Discussion

The targeted bi‐material structures exhibit large deformation amplitudes (up to +10%) with coefficients of thermal expansion greater than 10000 ppm/°C. Such unusual mechanical performance guided by a theoretical analysis based on analytical calculations and Finite Element Analysis simulations stems from the success of the challenging preparation of 3D printing composite filaments implemented for the rational design of 4D‐printed mechanical metamaterials.

### 2.1 Additive Manufacturing of SCO@polymer Composites

**FIGURE 1:**
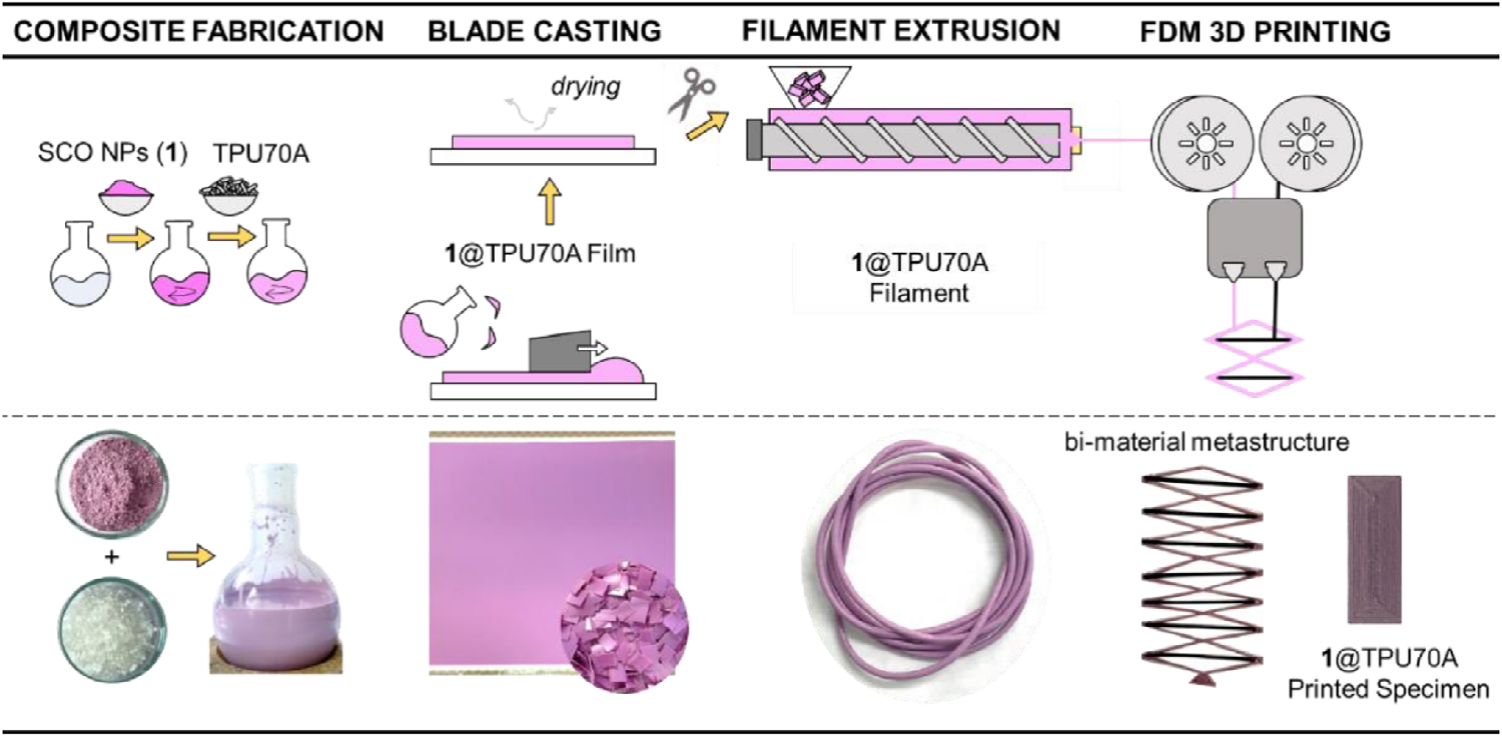
Scheme of the manufacturing process to obtain 4D printed SCO@TPU structures.

The primary bottleneck in our endeavor was to ensure the 3D printability of SCO composite materials with ca. 30% active fillers to optimize the strain [46], while preserving their intrinsic properties. Previous efforts have focused on stereolithography (SLA) 3D printing and provided high‐resolution printed objects, which effectively retained the SCO properties [51, 52]. However, the particle loads were limited by the increasing viscosity of the resin, hindering flow, and thus the printing of the subsequent layers. Notably, in dual‐material SLA printing, the charge was limited to less than 15 wt.%. Another interesting approach, using microfluidics‐based printing has been recently proposed, but neither the particle concentration, nor the printing quality can meet our requirements [53]. We have therefore developed a different 3D‐printing approach, based on fused deposition modelling (FDM). In the present context, the main advantage of FDM is that the flow is facilitated by the melting of the polymer matrix, allowing thus higher particle fillings, but a match between the melting temperature and the thermal stability of the particles must be found. After extensive experimental testing, TPU‐based **1**@TPU70A filaments with 30 wt.% SCO particles, suitable for FDM printing, were produced via a hot‐melt extrusion process using a single‐screw extruder (see the Supporting Information, for more details about sample fabrication and characterization). The extruded filaments were collected on a rotating spool and were dried to improve print quality. Finally, the filament was fed into an FDM printer. The brass nozzle diameter was 0.4 mm, and we printed layers with a height of 0.3 mm. Print speed, nozzle, and bed temperatures were carefully adjusted to ensure dimensional accuracy and layer adhesion, particularly important for soft or functional filaments. The scheme of the full manufacturing process, together with the photos of the casted film, extruded filament, and a printed specimen are shown in Figure 1 (see also Schemes S1 and S2).

**FIGURE 2:**
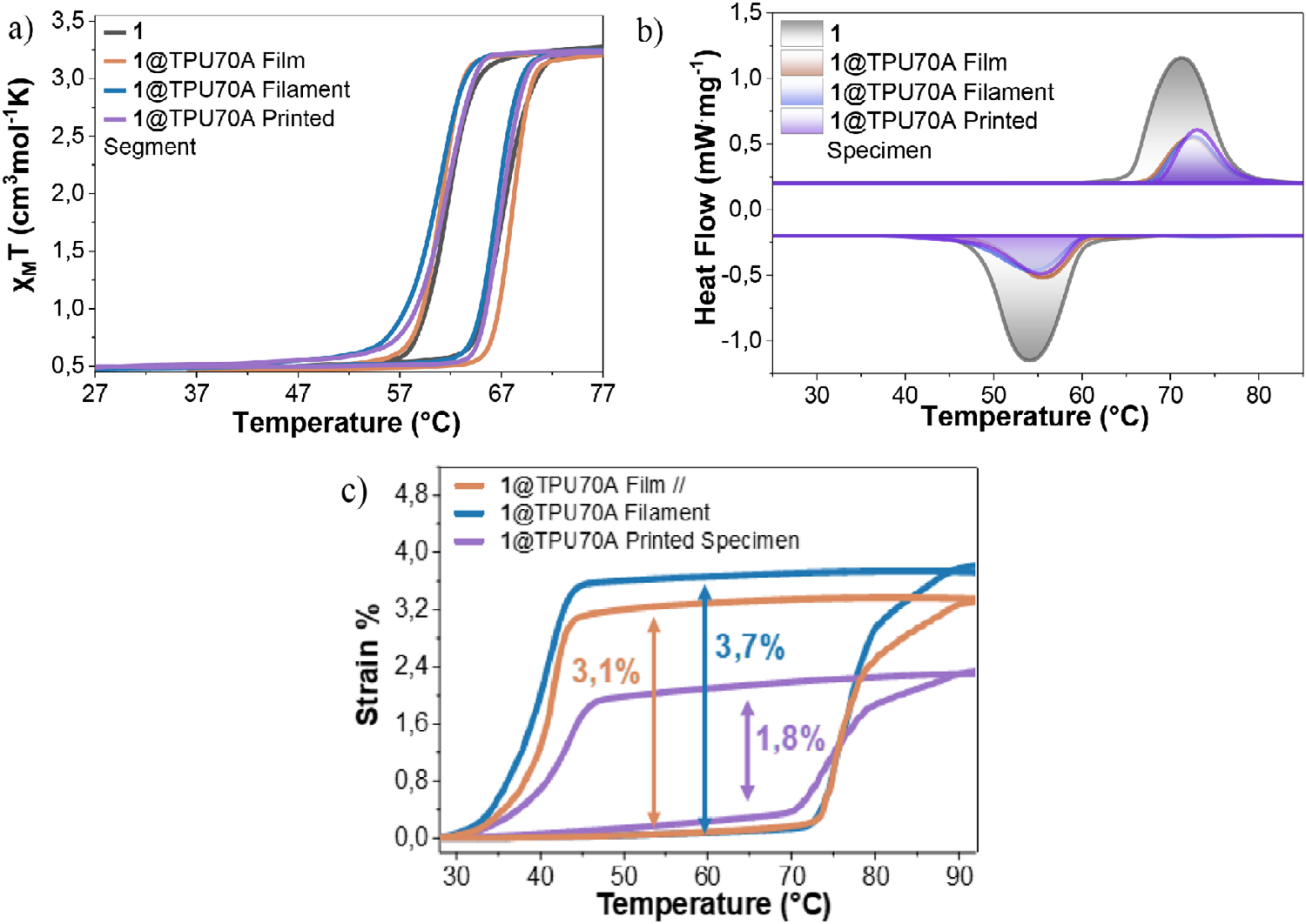
Temperature dependence of (a) the χ_M_ *T* product, (b) the heat flow (DSC), and (c) the strain for the SCO NPs, SCO@TPU70A film, SCO@TPU70A filament, and SCO@TPU70A printed segment/specimen.

The SCO properties of the **1**@TPU70A composites were assessed by variable temperature optical reflectivity (Figure S1), magnetometry (Figure 2a; Figure S2), and calorimetry (Figure 2b, see also Figure S3) measurements, showing comparable SCO properties for the crystalline powder and the composites at each stage of the fabrication process (see also Table 1 and Tables S1 and S2). By comparing the enthalpy change associated with the spin transition, calorimetry also allowed us to assess the effective particle load in the composites, which closely matched the targeted 30 wt.% (ca. 18 vol.%), demonstrating that both the particle concentration and the SCO properties were preserved during the whole fabrication process (Table 1). To assess the thermomechanical properties of the samples, dynamic uniaxial tensile studies were performed. As it can be expected, the **1**@TPU70A composites showed significant reinforcement of the storage modulus (E’) relative to the pure TPU70A, because of the embedment of the stiff SCO particles into the rather soft TPU70A matrix (Table 1). Temperature dependence of the mechanical properties, reported in Figures S4 and S5, mirrors trends previously reported for SCO composite films with other TPU grades with different Shore A hardness [46, 47].

**TABLE 1:** Effective particle concentration, storage modulus (E’), loss tangent (tanδ), transformation strain (ε), peak and average CTE of the **1**@TPU70A composites, and that of the neat polymer filament. * The specimen has a rectangular parallelepiped shape of 29 × 8 × 0.8 mm^3^.

Sample	Particle wt%	E’_30°C_ (MPa)	tanδ	Ε (%)	CTE^peak^ (ppm/°C)	CTE^ave^ (ppm/°C)
**1**@TPU70A film	29	23	0.074	3.1	5000	940
**1**@TPU70A filament	30	36	0.094	3.7	6000	1050
**1**@TPU70A printed specimen*	31	18	0.110	1.8	3000	640
TPU70A filament	0	7	0.050	N/A	130	130

Concerning our targeted application, of paramount importance is the ability of the **1**@TPU70A composites to produce a high transformation strain at the spin transition. To achieve that, we used engineered high aspect‐ratio, rod‐shaped SCO particles of **1** (see Figure S6), which are known to develop most of the transformation strain along their long axis and are prone to achieve preferential orientation in this direction [48]. As shown in Figure 2b (see also Figures S7 and S8), upon switching from the LS to the HS state (heating), an abrupt elongation is observed for each composite, a process that is reversible on cooling.

**FIGURE 3:**
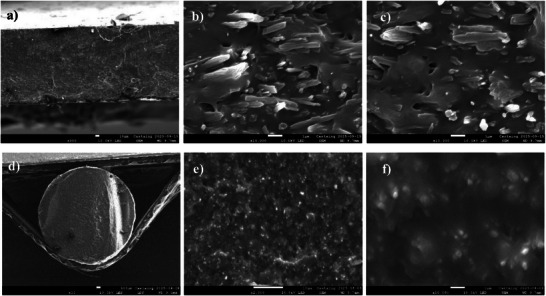
SEM images of the cross‐section of (a–c) the **1**@TPU70A film (cut perpendicular to the blade‐casting direction) and (d–f) the **1**@TPU70A filament at different magnifications.

The transformation strain associated with the SCO phenomenon changes at the successive fabrication steps, primarily because of the different particle orientations. (N.B. The change of mechanical properties might also impact this property.) This can be clearly seen in mechanical measurements conducted in different sample orientations (Figure S7) as well as in the scanning electron microscopy (SEM) images of the composites shown in Figure 3, in which the rather homogeneous dispersion of the particles is also evident both in the **1**@TPU70A film and **1**@TPU70A filament.

The results indicate that, compared to the blade‐casting technique used for film fabrication, the extrusion process promotes a more pronounced alignment of the particles along the flow direction, mostly due to the higher shear stress generated at the nozzle die during the filament production. On the other hand, the lower transformation strain in the printed object reveals a more random particle orientation within the polymer matrix.

### 2.2 Metastructure Design for Programmable CTE

**FIGURE 4:**
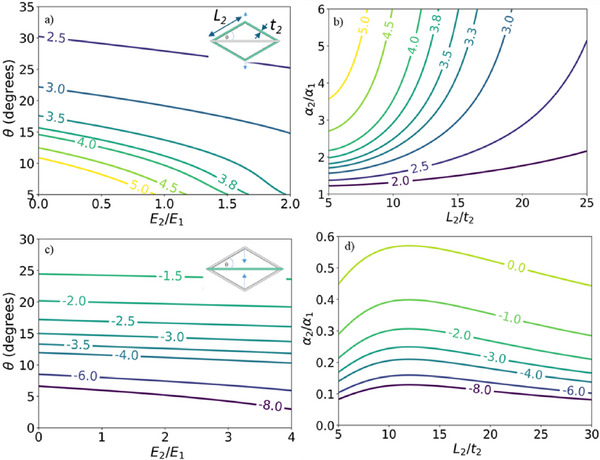
Analytical solutions of the CTE magnification factors *M_f_* (a, b) and $M_{f}^{N}$(c, d) using Equations (1) and Equation (10) in the SI, respectively (*M_f_* and $M_{f}^{N}$ values are given on each contour line). The structures corresponding to the positive and negative expansion cases are shown in the inserts with the colour code green for the ‘active’ SCO composite and grey for the "passive" material. The free simulation parameters are the internal angle θ, the Young's modulus ratio $\frac{E_{2}}{E_{1}}$, the CTE ratio $\frac{\alpha_{2}}{\alpha_{1}}$, and the aspect ratio $\frac{L_{2}}{t_{2}}$. The fixed simulation parameters are gathered in Table S3.

In the literature, numerous bi‐material cellular structures have been described to tune and amplify CTEs [13, 14, 15, 16, 17, 18, 19, 20, 21, 22]. To demonstrate our 4D‐printing concept, we have chosen a bi‐material diamond cell, described in ref. [18] and shown in the inset of Figure 4a (see also Scheme S3). When subjected to a thermal stimulus, the mismatched material properties give rise to internal stress fields, resulting in either positive (expansive) or negative (contractive) motion, depending on the placement of the lower CTE "passive" and higher CTE "active" materials within the structure (Figure 4a vs. c). It is important to note that this structure does not provide the highest known CTE amplification, but it has a clear deformation mechanism, which can be investigated using straightforward analytical and numerical approaches. Initially, we conducted parametric studies with the aim to elucidate the effect of cell geometry and material properties on the thermal expansion behavior of the targeted diamond metastructure. These studies were conducted using an analytical approach described in Section S3. The model defines a thermal magnification factor, *M_f_*, representing the amplification of the CTE within the bi‐material structure with respect to the neat SCO composite. The derived expression (Equation (1)) captures the effect of Young's modulus ratio $\;\frac{E_{2}}{E_{1}}$ and thermal expansion ratio $\frac{\alpha_{2}}{\alpha_{1}}$ of the two materials used for the construction (subscript 2: hypotenuse, subscript 1: base), as well as that of the internal strut angle θ and other geometrical/material parameters, which are defined in the Supporting Information.

(1)

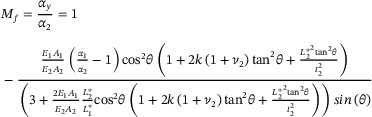




Figure 4 summarizes the key findings through a series of contour plots for different design configurations, whereas the input simulation parameters are gathered in Table S3. In Figure 4a, *M_f_* is plotted as a function of stiffness ratio $\frac{E_{2}}{E_{1}}$ and internal angle θ, assuming the SCO composite occupies the hypotenuses. The results indicate that large deformation is achieved when θ is small and when the active material gets softer with respect to the passive component. Nevertheless, the latter effect is not pronounced, the variation of *M_f_* against $\frac{E_{2}}{E_{1}}$ being rather smooth in the investigated parameter range. In Figure 4b, the interplay between the CTE ratio ($\frac{\alpha_{2}}{\alpha_{1}}$) and the aspect ratio of the hypotenuse (length/thickness, $\frac{L_{2}}{t_{2}}$) is elucidated. As it can be expected, the increase of thermal expansion ratios leads to an increase of the magnification factor. However, at high ratios ($\frac{\alpha_{2}}{\alpha_{1}}>∼5$) the effect of this parameter tends to disappear. On the contrary, the effect of $\frac{L_{2}}{t_{2}}$ is substantial with lower values leading to higher *M_f_*. These results highlight geometry as the primary lever for tailoring actuation—even if material property contrasts must be also taken into account.

We have also examined the inverted configuration, where the SCO composite is placed in the inner struts and the passive material in the hypotenuses. This reverses the actuation direction, yielding negative magnification factors ($M_{f}^{N}$). The simulation results shown in Figure 4c,d reveal that similar design principles apply as for the case of positive magnifications, but the mismatch of material properties may have a higher impact in this case. This inversion capability demonstrates the bidirectional programmability of the system via material placement alone. Interestingly, Figure 4d indicates that for specific parameter values one can also achieve zero thermal expansion, although this application does not require the use of an SCO material.

Based on these preliminary results, we have decided to use as inactive material the TPU grade TPU98A, which affords for the desired material contrasts. (See Table S3 and Figure S9 for the measured mechanical properties for TPU98A.) It is important to underline that using a “passive” material with higher stiffness and lower CTE than TPU98A is not expected to bring considerable improvement of the magnification factor and the choice of TPU98A was thus largely motivated by its similar composition with TPU70A, ensuring good adhesion between them. Using these two materials, the final design was made by considering the lowest possible θ and $\frac{L_{2}}{t_{2}}$ values, while preserving the printability of the structure. To predict more accurately the magnification factor and to assess the internal stress distribution under thermal load we conducted finite element analysis (FEA) using the commercial software COMSOL (see details in the Supporting Information).

**FIGURE 5:**
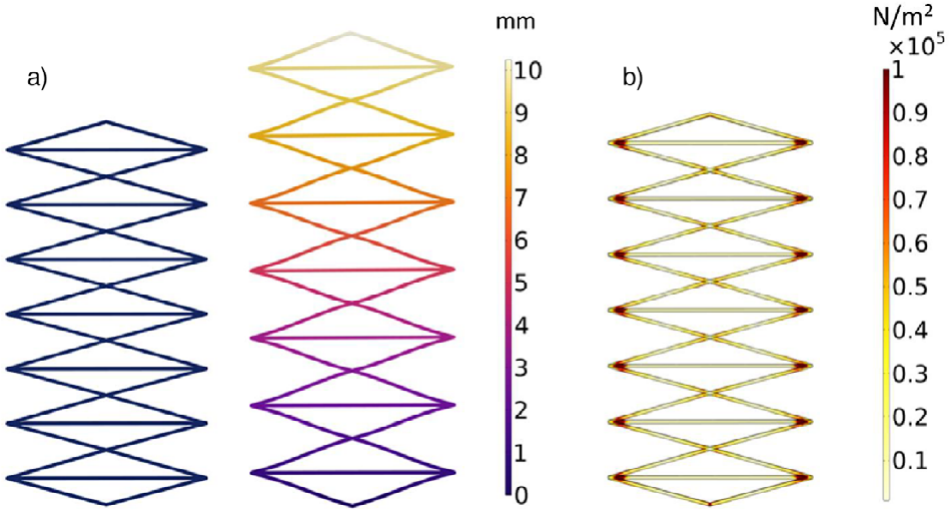
Finite element simulation of (a) the deformation of the designed positive CTE metastructure when switched between the LS (left) and HS (right) spin states, and (b) the Mises stress distribution in the HS state.

The displacement shown in Figure 5a and Figure S10 predicts magnification factors of ca. +10 and −8 for the positive and negative expansion cases, respectively, when going from the low to the high spin state. As it can be expected, the Mises stress induced in the struts upon heating remains low, except in the vicinity of the bonded joints where thermal misfits and bending produce stress concentrations. Nevertheless, the calculated peak values (ca. 0.1 MPa) should remain below the yield stress, i.e., no extensive plastic flow is expected in the operating temperature range.

### 2.3 Deformation of the Printed Metastructures

**FIGURE 6:**
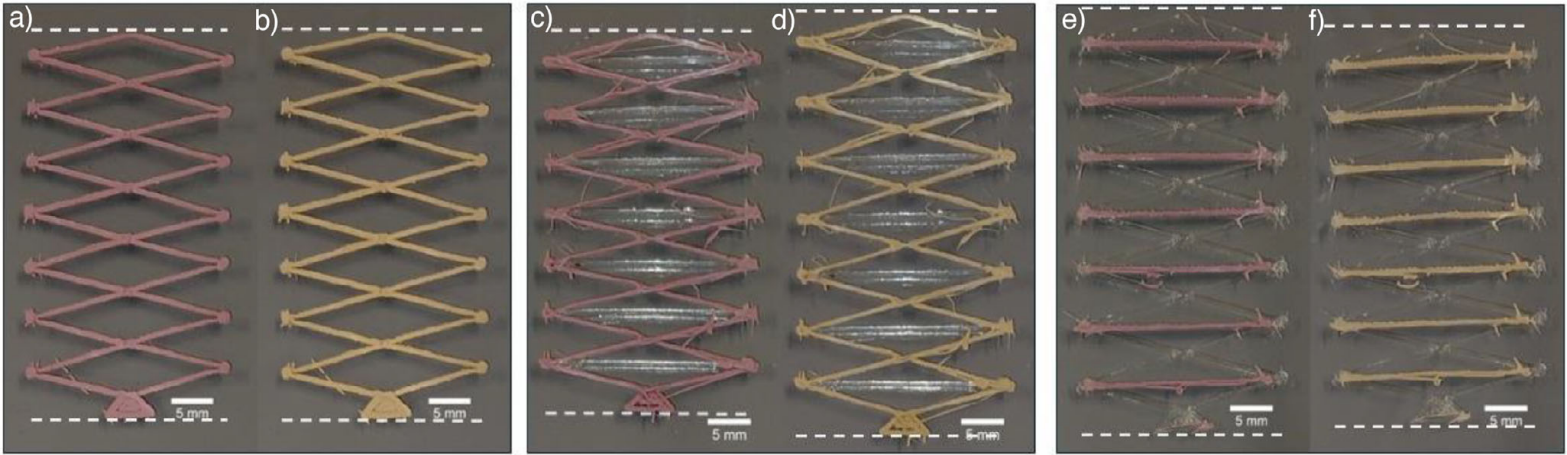
Photographs of a structure without the TPU98A base at (a) 25°C and (b) 95°C. Photographs of a positive CTE bi‐material metastructure (white: TPU98A, pink/yellow: **1**@TPU70A) recorded at (c) 25°C and (d) 95°C. Photographs of a negative CTE bi‐material metastructure (white: TPU98A, pink/yellow: **1**@TPU70A) at (e) 25°C and (f) 95°C.

To validate the theoretical predictions and assess the performance of the SCO‐based metastructures, we fabricated a series of architected lattices using a dual‐material FDM printer. The dual extrusion module allows printing alternately either a TPU98A or a **1**@TPU70A composite filament, according to the target structures. Figure 6c,d shows the thermally induced deformation of a single column (1D) structure, programmed for positive CTE. When the structure is heated from room temperature to ca. 60°C (Figure S11) the constituent materials display only ordinary thermal expansion with $\frac{\alpha_{2}}{\alpha_{1}}=0.6$, which corresponds to a magnification factor close to zero (see Figure 4d). The resulting minor contraction, not perceptible by eye, characterizes a 3D‐printed metastructure, constructed from “passive” materials. When the structure is further heated to ca. 95°C, the hypotenuses change color from pink to yellowish due to the thermally‐induced spin transition (Figure 6d). In parallel, it exhibits a large (ca. 10.5%), well‐perceptible deformation in the direction normal to the “passive” base, thus demonstrating the huge gain obtained by using an active material. Obviously, the CTE of this 4D‐printed structure is not constant, but depends on the temperature range considered. For the relevant temperature range of 60°C–95°C, the average CTE^ave^ is ca. +3860 ppm/°C, whereas its peak value near the spin transition temperature reaches CTE^peak^ = +14400 ppm/°C. These values exceed by 1–2 orders of magnitude the CTE of similar bi‐material diamond structures constructed with “passive” materials [18]. It is important to underline that the active material alone, in the absence of the passive base, produces only a relatively small deformation of ∼1% (Figure 6a,b). In other words, it is the synergistic combination of the active material with the dual‐material cellular architecture, which affords for the spectacular deformation of the 4D‐printed structure. The ratio of deformation between the single‐ and bi‐material structures allows us to assess a magnification factor of *M_f_* = 10.5, in agreement with the prediction of FEA simulations. One should note, however, that the good match between FEA and experiment concerns only the ratio $\frac{\alpha_{y}}{\alpha_{2}}$, whereas the absolute value of α_*y*_ is overestimated in the simulations due to the lack of accuracy of certain input parameters (α_*i*_, E_i_) and the imperfect printing quality.

**FIGURE 7:**
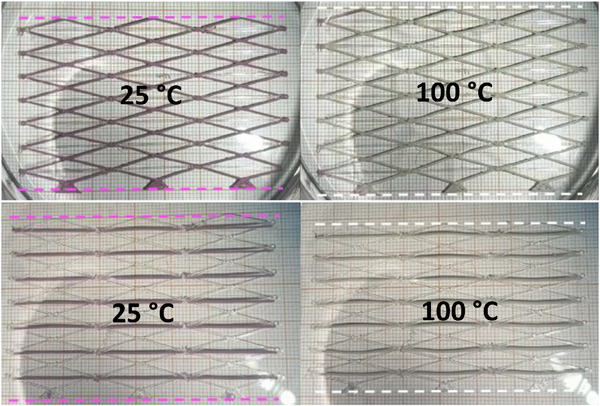
Photographs recorded at 25°C and 100°C of two 2D lattices programmed for positive (top) and negative (bottom) expansion.

The above discussed giant actuation was repeatedly observed for the same device and also for similar ones with different material configurations and different structures (see Figures 6 and 7 and Figures S12 and S13). Notably, Figure 6e,f shows photographs for an ‘inverted’ structure, wherein the material roles are reversed, with the SCO phase integrated into the base and the TPU98A into the hypotenuses. In this case, the structure contracts upon heating, displaying a reversible displacement of −7.7%. Accordingly, the calculated magnification factor ($M_{f}^{N}=-7.7$) matches the value predicted by FEA, whereas CTE^ave^ and CTE^ave^ for this inverted structure can be estimated as −2200 ppm/°C and −10600 ppm/°C, respectively.

Figure S13 also demonstrates that the thermally‐induced deformation is reversible across multiple thermal cycles, except for the first heating, due to a run‐in effect corresponding to the release of solvent and the stabilization of the crystallinity of the networks. This structural stability under repeated actuation confirms the absence of any substantial irreversible deformation and reveals robust adhesion between the composite materials.

Besides the single‐column (1D) lattices, we have also printed more extended 2D lattices (21 unit cells), programmed for positive and negative CTE. As shown in Figure 7, upon heating to 100°C, the structures exhibit perceptible, large strains along the vertical axis, reaching +7.3% (CTE^peak^ = +10000 ppm/°C) and −8.3% (CTE^peak^ = −11400 ppm/°C), respectively, which are reversible upon cooling. These 2D structures were printed using a second batch of **1**@TPU composite filament, displaying slightly different thermomechanical properties in comparison with the first batch employed for the fabrication of the 1D lattices. For this reason, a direct comparison of the magnification factors is not straightforward. Nevertheless, we can note that, in line with the predictions of the FEA simulations (see Figure S10), the 2D lattices appear to generate comparable (positive and negative) deformations as the 1D analogues.

## 3 Conclusions

In this paper, we report a versatile and programmable class of thermally actuated metamaterials by embedding spin crossover nanoparticles into architected dual‐material frameworks. Our approach synergistically combines material‐level phase transition with metamaterial design principles to create structures capable of reversible directional deformation in response to heating. Using an analytical model, by adjusting unit cell geometry and material properties, we can quickly explore the design space for tuning the magnitude and direction of motion—achieving positive and negative actuation using the same structural template. More accurate predictions can then be made using FEA simulations. An original and challenging preparation of filaments allows us to 3D print high‐quality composites of the phase‐change material by means of FDM, thus transforming the theoretical design into real‐world metastructures. The demonstrated structures exhibit large deformation amplitudes (up to +10% and down to −8%) and repeatable performance, underscoring their potential for use in soft robotics, intelligent textiles, and adaptive/reconfigurable structural systems. Remarkably, the accessible range of coefficients of thermal expansion spans between +14400 and −11400 ppm/°C, largely exceeding those previously reported for similar designs employing “passive” materials. The obtained performance is not an upper limit and could be improved by playing with the chemical composition and microstructure of the employed materials and/or by printing more advanced, Kirigami‐inspired metastructures [21]. Besides the enhanced thermal expansion, SCO based metamaterials display a range of remarkable properties, such as the change of color, dielectric permittivity, entropy, and elastic constants. This multi‐functionality combined with their versatility in terms of the nature (gradual, stepped, hysteretic) and the temperature range of the spin transition, opens up unprecedented possibilities to create smart architectures, able to perform a variety of functional tasks.

## Conflicts of Interest

The authors declare no conflicts of interest.

## Supporting information




**Supporting File**: adma73061‐sup‐0001‐SuppMat.pdf.

## Data Availability

The data that support the findings of this study are available in the supplementary material of this article.
