# Supplementary material for: 4D‐Printed Spin Crossover Metamaterials with Giant Programmable Positive or Negative Thermal Expansion
Source: Adv Mater. 2026 Apr 14;38(32):e22073. doi: 10.1002/adma.202522073 (PMC13244803; doi:10.1002/adma.202522073)

SUPPORTING INFORMATION

4D-Printed Spin Crossover Metamaterials with Giant Programmable Positive or Negative Thermal Expansion

Adelais Trapali, Yuteng Zhang, Seyed E. Alavi, Nagham Mawassy, Raja Zulkarnain, Gábor Molnár, Lionel Salmon*, Azzedine Bousseksou*

LCC, CNRS & Université de Toulouse (UPS, INP), 31077 Toulouse, France

* lionel.salmon@lcc-toulouse.fr, azzedine.bousseksou@lcc-toulouse.fr

Section S1. Experimental methods

Section S2. Sample characterization

Section S3. Theoretical analysis of the metastructures

Section S4. Experimental characterization of the metastructures

Section S1. Experimental methods

Sample fabrication. [$Fe(4-NH_2-1,2,4-triazole)_3]SO_4$ (**1**) spin-crossover nanoparticles (SCO NPs) were synthesized according to a previously described procedure (see J. Mater. Chem. C, 2020, 8, 6001). Two different synthesis batches have been used with different rod lengths (**Figure S6**), providing two composite batches, named 'Batch 1' and 'Batch 2', which were used for the printing of 1D and 2D lattices, respectively. Except otherwise mentioned, all figures and tables in the MS refer to 'Batch 1', whereas we provide characterization data for both batches in the SI. **1**@TPU70A composite films with a SCO NPs loading of 30% (wt.%) were obtained by blade casting as described by Zan and coworkers (see Adv. Intel. Syst. 2023, 5, 2200432). All reagents and solvents were purchased from Sigma-Aldrich and used as received without any further purification. TPU70A and TPU98A were supplied by Recreus and Dailyfil, respectively. **1**@TPU70A filaments, suitable for FDM printing, were produced via the hot-melt extrusion process using a Noztek Touch single-screw extruder **(Scheme S1**). Pre-cut pieces of a blade casted **1**@TPU70A film containing 30 wt% of **1** were introduced into the hopper and continuously fed into the extruder's barrel with a rotational frequency of 58 rpm at 170 °C. The resulting composite filament was extruded through a brass nozzle and collected on a rotating spool. The spool speed was carefully adjusted to maintain a consistent filament diameter of 1.75 ± 0.05 mm, which is required by the printer. Prior to printing, the filaments were dried in a filament dry box at 60 °C for at least 12 hours to minimize moisture-related issues such as stringing and improve print quality. Finally, the filament was fed into an FDM printer (Snapmaker 2.0, Snapmaker Inc.) shown on **Scheme S2**. The brass nozzle diameter was 0.4 mm, and the printed layers were of 0.3 mm in height. During printing, the nozzle temperature was set to 205 °C, while the bed temperature was maintained at 60 °C. The printing speed was at 10 mm/s to ensure dimensional accuracy and layer adhesion. All structures were printed without additional support materials, and no chamber heating was applied during the printing process.

Sample characterization. Variable-temperature optical reflectivity studies were carried out at a heating/cooling rate of ± 2°C·min^{-1} using a MOTIC SMZ-168 microscope equipped with a MOTICAM 1000 color CMOS camera. Differential scanning calorimetry (DSC) measurements were realized with a 3500 Sirius DSC-NETZSCH instrument at a temperature sweep rate of ± 10 °C·min^{-1} under a N_2 gas purge flow of 20 mL·min^{-1}. Temperature and heat flow were calibrated using the melting transition of indium. Magnetic susceptibility data were acquired with a Quantum Design MPMS5 magnetometer at an applied magnetic field of 0.1 T with ± 2 K·min^{-1} heating/cooling rates, and they were corrected for diamagnetic contributions. Dynamical mechanical analysis (DMA) experiments were conducted with a DMA850 (TA Instruments) apparatus, in uniaxial tension configuration, at an oscillation frequency of 1 Hz, oscillation strain amplitude of 0.08 %, a temperature rate of ± 3 °C·min^{-1} and an applied 0.1 N preload force that was continuously adjusted proportionally (150%) to the change of the sample stiffness to ensure that the dynamical force remains inferior to the static force throughout the course of the experiment. For the film samples, DMA measurements were realized on specimens cut parallel (//) and perpendicular (⊥) to the blade casting direction. TEM images of **1** were collected using a JEOL JEM 1400 Rio operated at 120 kV. Samples for TEM were prepared by suspending **1** in absolute EtOH and subsequently placing a drop of the suspension on copper grid coated with carbon. SEM images were acquired using a JEOL JSM 7800 F Prime operated at 5 kV. Samples for SEM were prepared by breaking the **1**@TPU70A films and **1**@TPU70A filaments at liquid nitrogen and metallizing the cross-section with Pt. Printed metastructures were immersed in an oil-bath and their thermal deformation was recorded.

Scheme S1. Schematic representation of the filament extrusion process. The extruded **1**@TPU filament is collected on a spool, which can be used in the 3D printer. Note the color change of the filament from white (near the extruder nozzle) to pink (on the spool), due to the change of its temperature and, consequently, its spin state.

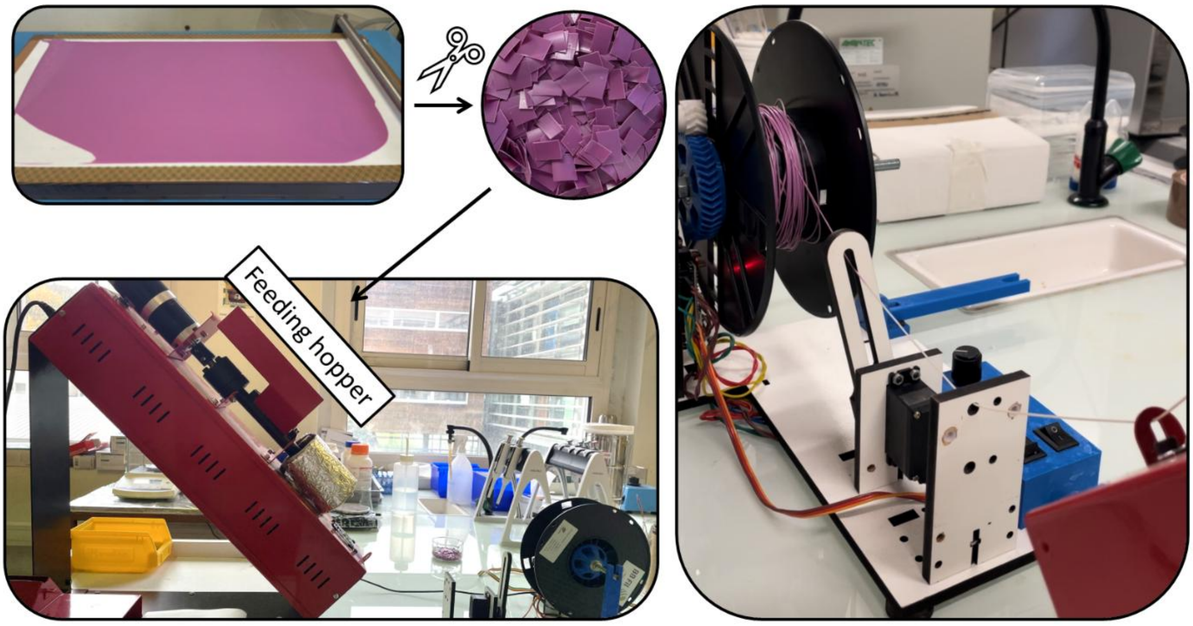

Scheme S2. Schematic representation and photos of the dual material FDM printing. Pictures of the SCO-based composite are also shown right after printing with bed temperature of 60°C and at room temperature.

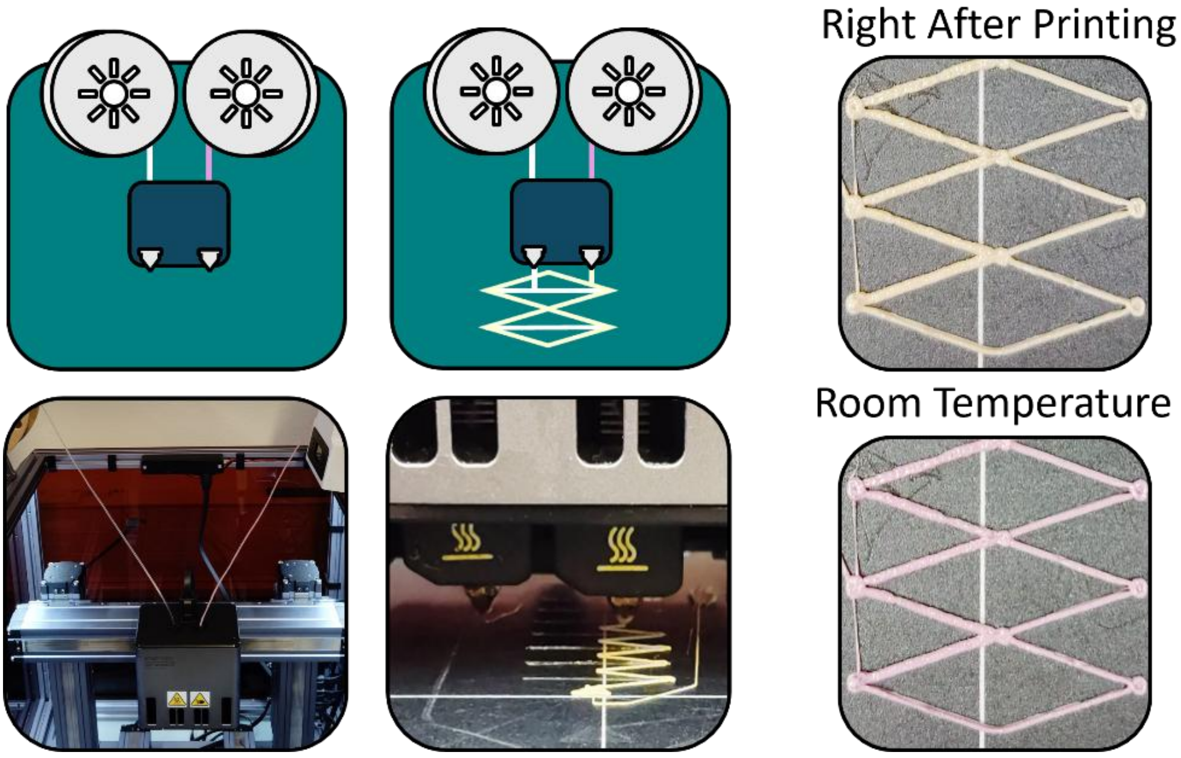

Section S2. Sample characterization

Figure S1. Variable temperature optical reflectivity studies for **1** (grey), **1**@TPU70A film (orange), **1**@TPU70A filament (blue) and **1**@TPU70A printed objects (purple) for (a) batch 1, and (b) batch 2. Heating/cooling rates: ± 2 °C·min^{-1}

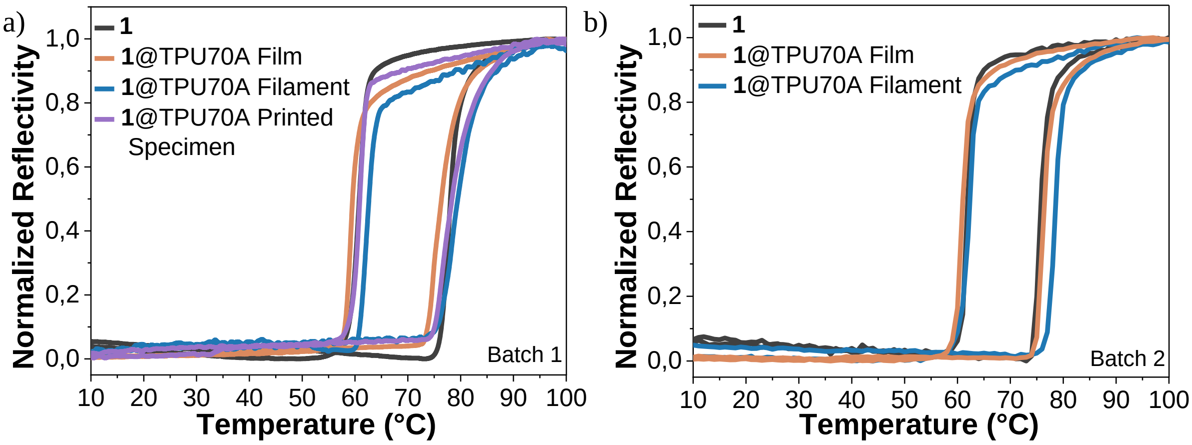

Figure S2. Temperature dependence of the χ_MT product for the SCO NPs (grey line), SCO@TPU70A film (orange), SCO@TPU70A filament (blue), and SCO@TPU70A printed segment/segment (purple) for (a) batch 1, and (b) batch 2. The data shown correspond to the third thermal cycle. χ_M stands for the molar magnetic susceptibility. Heating/cooling rates: ± 2 K·min^{-1}

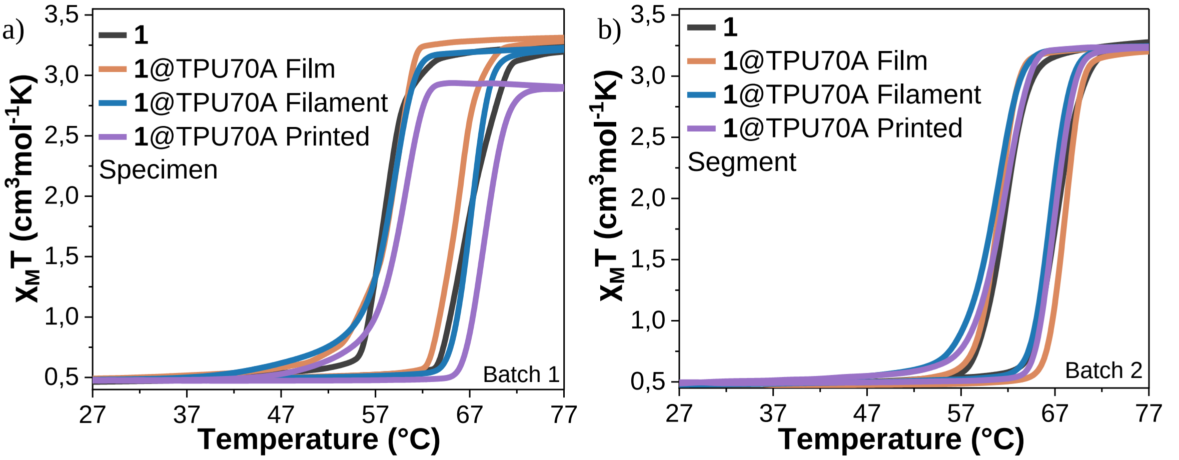

Figure S3. Differential Scanning Calorimetry (DSC) of **1** (grey), **1**@TPU70A film (orange), **1**@TPU70A filament (blue), and **1**@TPU70A printed specimen (purple) for a) batch 1, and b) batch 2. Heating/cooling rates: ± 10 °C·min^{-1}

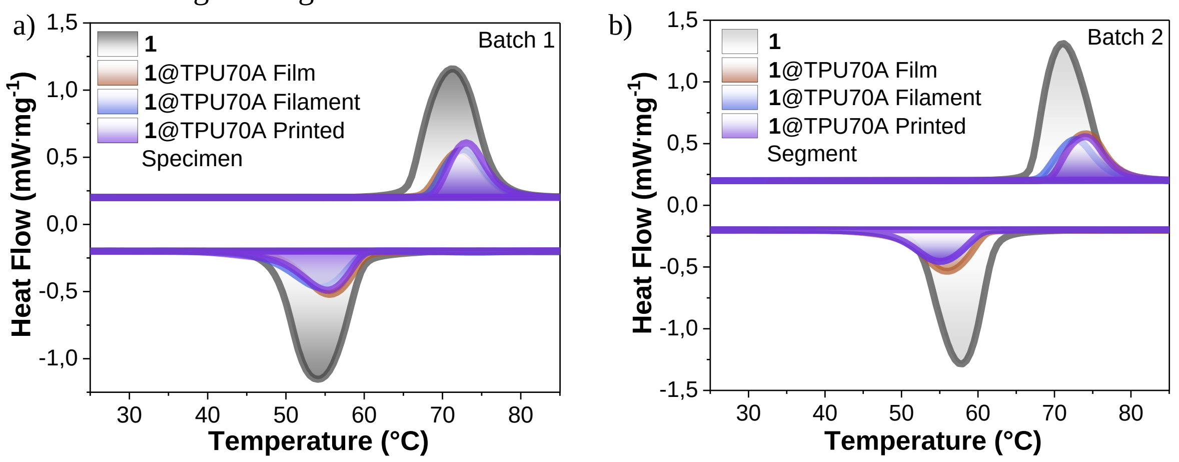

Table S1. Spin transition temperatures for SCO NPs and **1**@TPU70A composites extracted from optical reflectivity, DSC, and magnetic susceptibility measurements for batch 1 and batch 2. Differences between the methods are observed due to the different temperature scan rates and different sample holders used, leading to thermal lags between the temperature measured by the sensor and the actual sample temperature. The 'real' transition temperatures are best reflected by the magnetic measurements. Slight shift of transition temperatures could be explained by residual solvents or other defects.

		**Optical reflectivity**			**DSC**			**Magnetic susceptibility**		
	**Sample**	T↑	T↓ (°C)	ΔT	T↑	T↓ (°C)	ΔT	T↑	T↓ (°C)	ΔT
Batch 1	**1**	78	61	17	71	54	17	67	59	8
	**1**@TPU70A Film	75	59	16	72	56	16	66	58	8
	**1**@TPU70A Filament	79	63	16	73	54	19	67	59	8
	**1**@TPU70A Printed Specimen	77	61	15	73	55	18	68	60	8
Batch 2	**1**	76	62	14	71	58	13	67	60	7
	**1**@TPU70A Film	76	61	15	74	56	18	68	61	7
	**1**@TPU70A Filament	78	62	16	73	55	18	67	61	6
	**1**@TPU70A Printed Segment	72	64	8	74	55	19	67	61	7

Table S2. Enthalpy variation (ΔH) associated with the spin transition obtained for SCO NPs and **1**@TPU70A composites from DSC measurements. Targeted and effective SCO NPs loadings are also noted. SCO NPs density at LS state = 2.086 g/cm^3 (taken from Cryst. Growth Des. 2023, 23, 3, 1903–1914), TPU70A density = 1.08 g/cm^3 (provided by Recreus). The targeted particle concentration is effectively achieved in each composite sample for both synthesis batches without any degradation of the SCO properties. With asterisk (*) is denoted the SCO NPs concentrations calculated from ICP-AES analysis of the Fe content.

	**Sample**	**Targeted (wt. %)**	**ΔH ($J \cdot g^{-1}$)**	**Effective (wt. %)**	**Effective (vol. %)**
Batch 1	**1**	N/A	44.6	100	N/A
	**1**@TPU70A Film	30	13.7	31	19
	**1**@TPU70A Filament	30	13.4	30 (29*)	18
	**1**@TPU70A Printed	30	14.0	31	19
Batch 2	**1**	N/A	44.0	100	N/A
	**1**@TPU70A Film	30	14.4	33	20
	**1**@TPU70A Filament	30	12.3	28 (30*)	17
	**1**@TPU70A Printed	30	13.5	31	19

Figure S4. Temperature dependence of the storage modulus (E') for the **1**@TPU70A filament (blue), **1**@TPU70A film (orange) cut in parallel to the blade casting direction (//), and the commercially available TPU70A filament (bottom) for a) batch 1, and b) batch 2. Heating (line), and cooling (short dot) refer to the second thermal cycle. Heating/cooling rates: ± 3 °C·min^{-1}. A softening is observed consistently near the spin transition temperatures due to anelastic relaxations associated with the spin transition.

a)

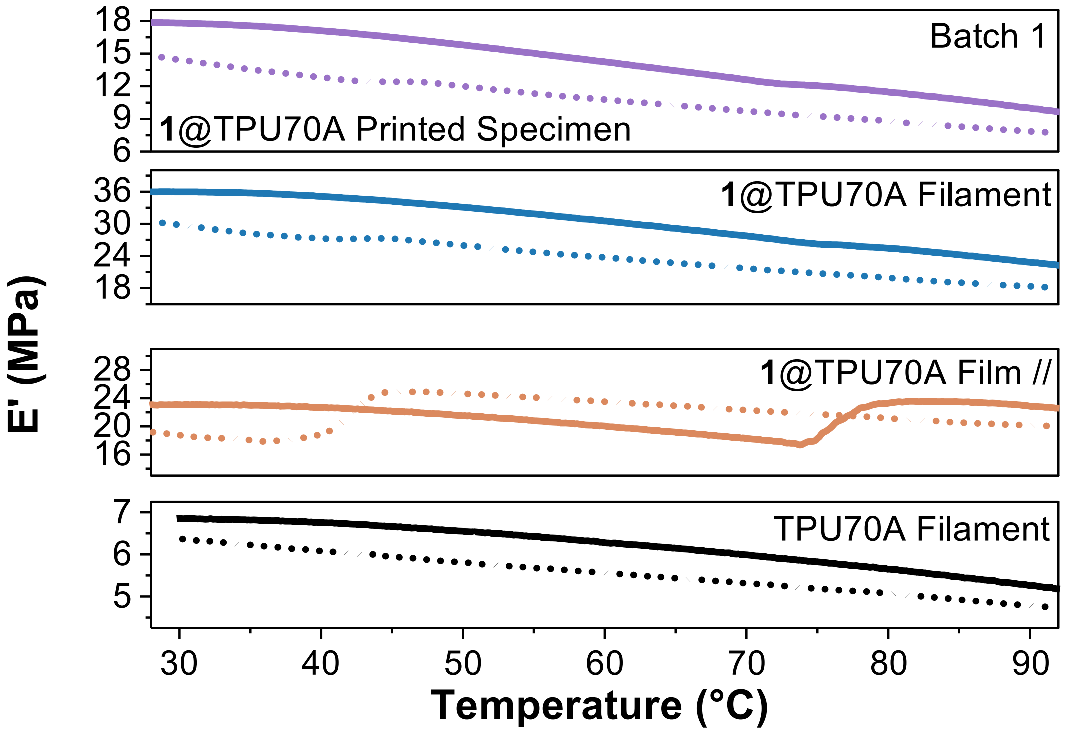

b)

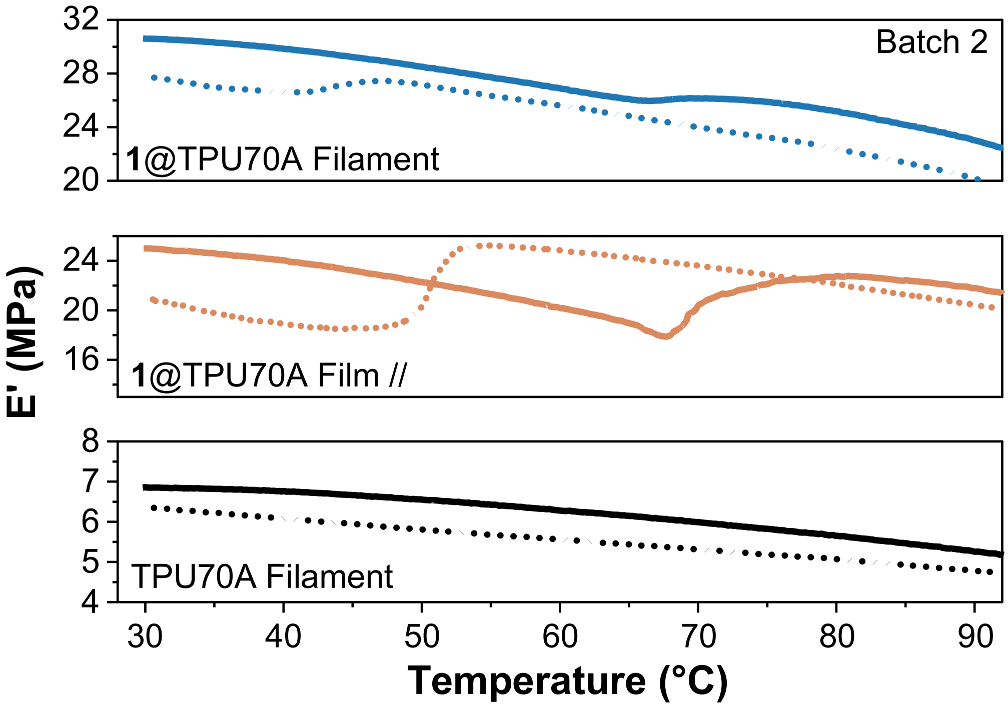

Figure S5. Temperature dependence of the loss modulus (E'') for the **1**@TPU70A filament (top), **1**@TPU70A film (middle) cut in parallel to the blade casting direction (//), and commercially available TPU70A filament (bottom). Heating (line), and cooling (short dot) refer to the second thermal cycle. Heating/cooling rates: ± 3 °C·min⁻¹. A dissipation peak is observed consistently at the spin transition due to anelastic relaxations associated with the spin transition.

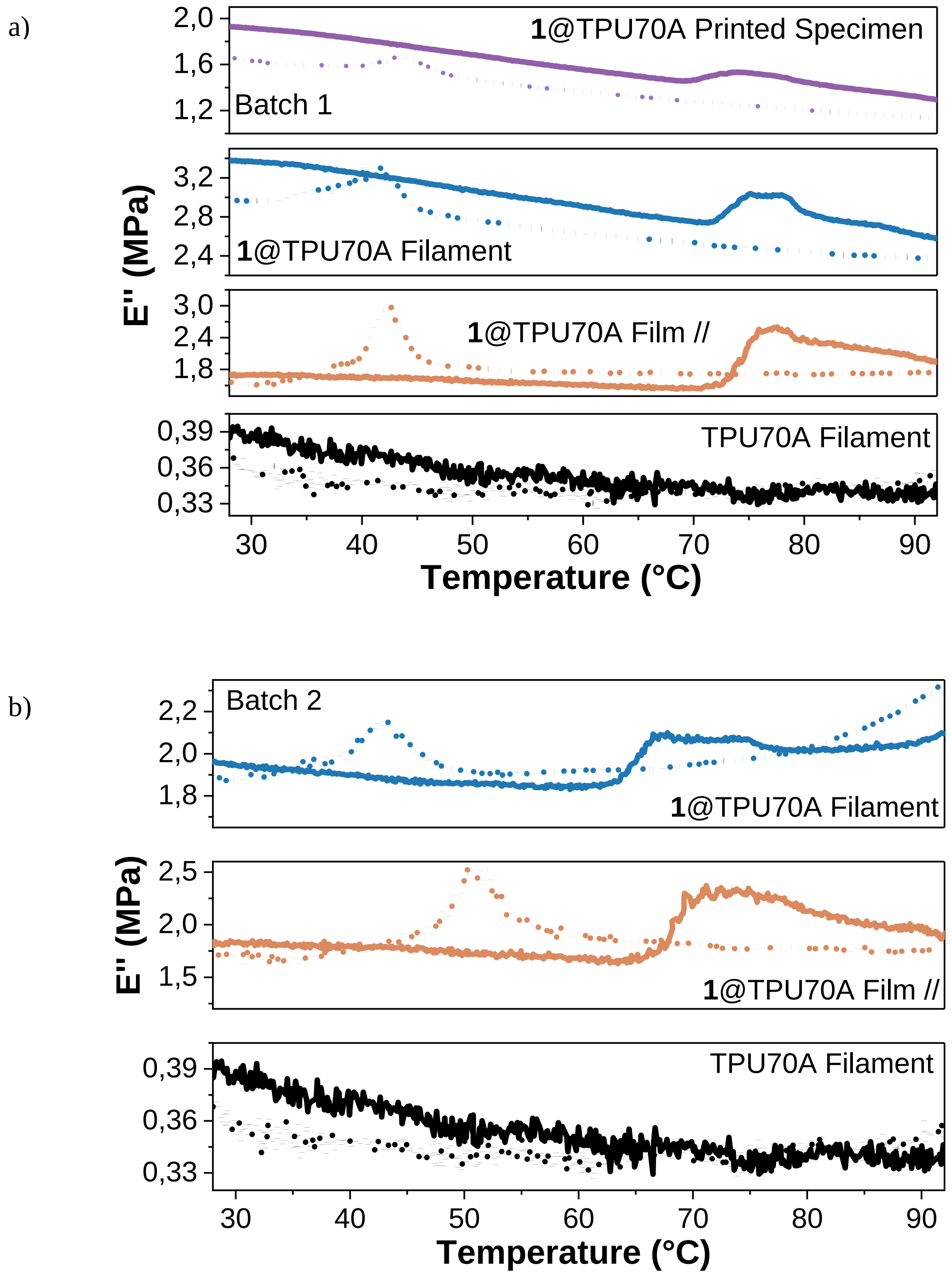

Figure S6: Representative transmission electron microscopy images of the SCO particles for a) batch 1, and b) batch 2.

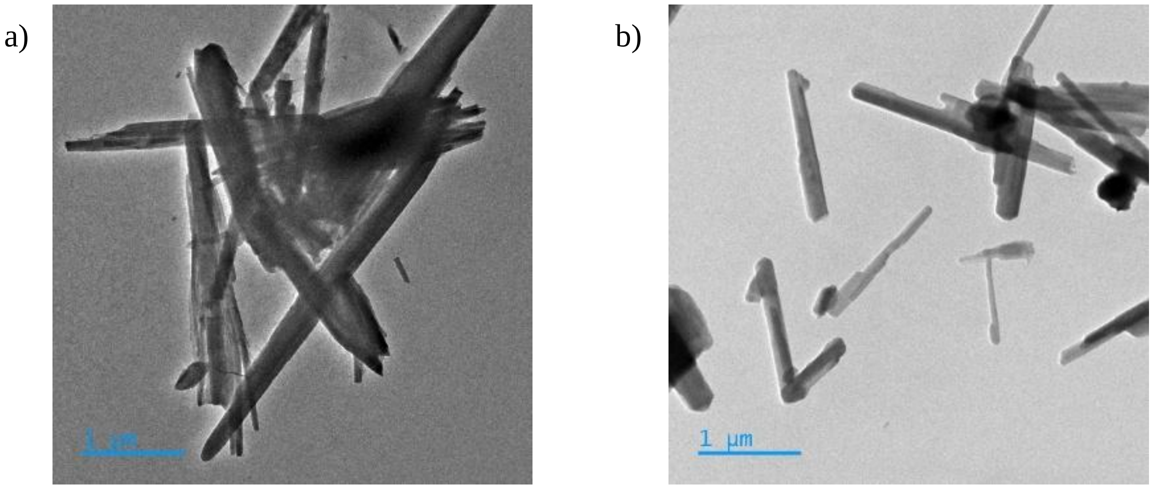

Figure S7. Temperature dependence of the thermal strain expressed in % for the **1**@TPU70A film cut either parallel (//, orange) and perpendicular (⊥, green) to the blade casting direction and that of the **1**@TPU70A filament (blue) and **1**@TPU70A printed object (purple) for a) batch 1, and b) batch 2. The curves refer to the second thermal cycle. Heating/cooling rates: ± 3 °C·min^{-1}. The pronounced difference between the parallel and perpendicular cut film samples derived from batch 1 indicates preferential particle orientation along the parallel direction. The transformation strain is always the highest in the filaments, denoting preferential particle orientation induced by the extrusion process.

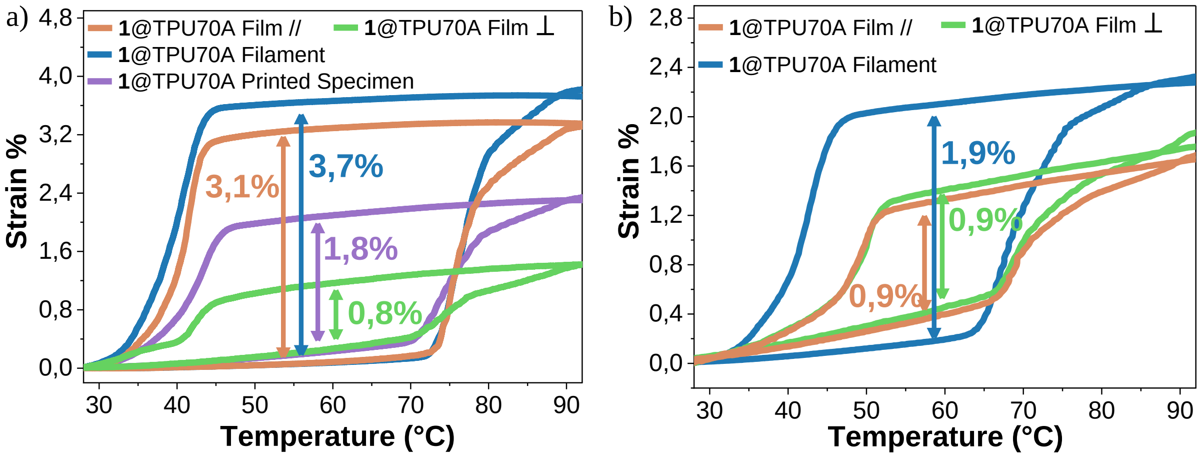

Figure S8. Temperature dependence of CTE obtained from the temperature derivatives of the curves shown in **Fig. S7** for the **1**@TPU70A film cut parallel (//, orange) and perpendicular (⊥, green) to the blade casting direction and that of the **1**@TPU70A filament (blue) and **1**@TPU70A printed object (purple) for (a) batch 1, and (b) batch 2.

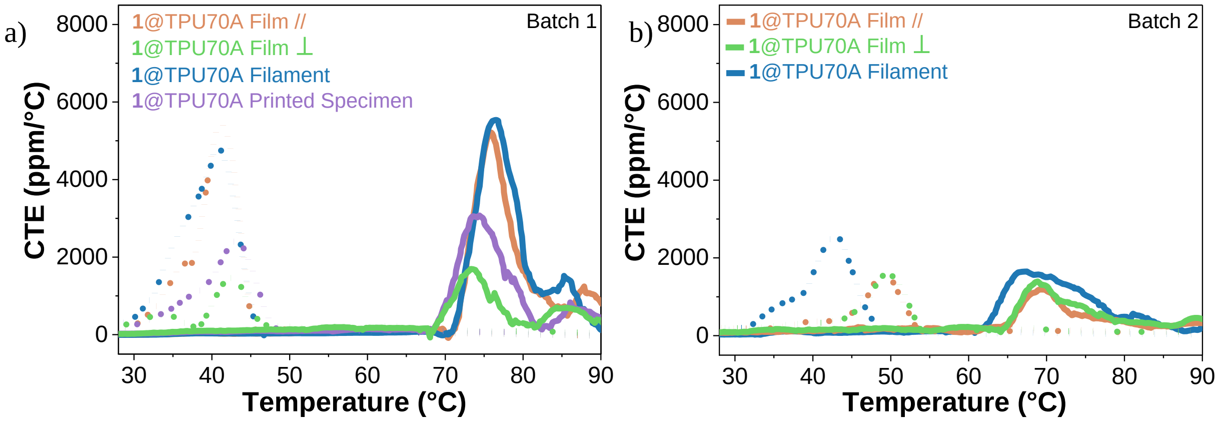

Figure S9. Thermomechanical characterization of a 3D-printed TPU-98A sample using DMA: temperature variation of (a) storage modulus (E’), (b) strain expressed in %, and (c) CTE. The curves correspond to the second thermal cycle, with red and blue colors indicating the heating and cooling. Heating/cooling rates: ± 3 °C·min^{-1}.

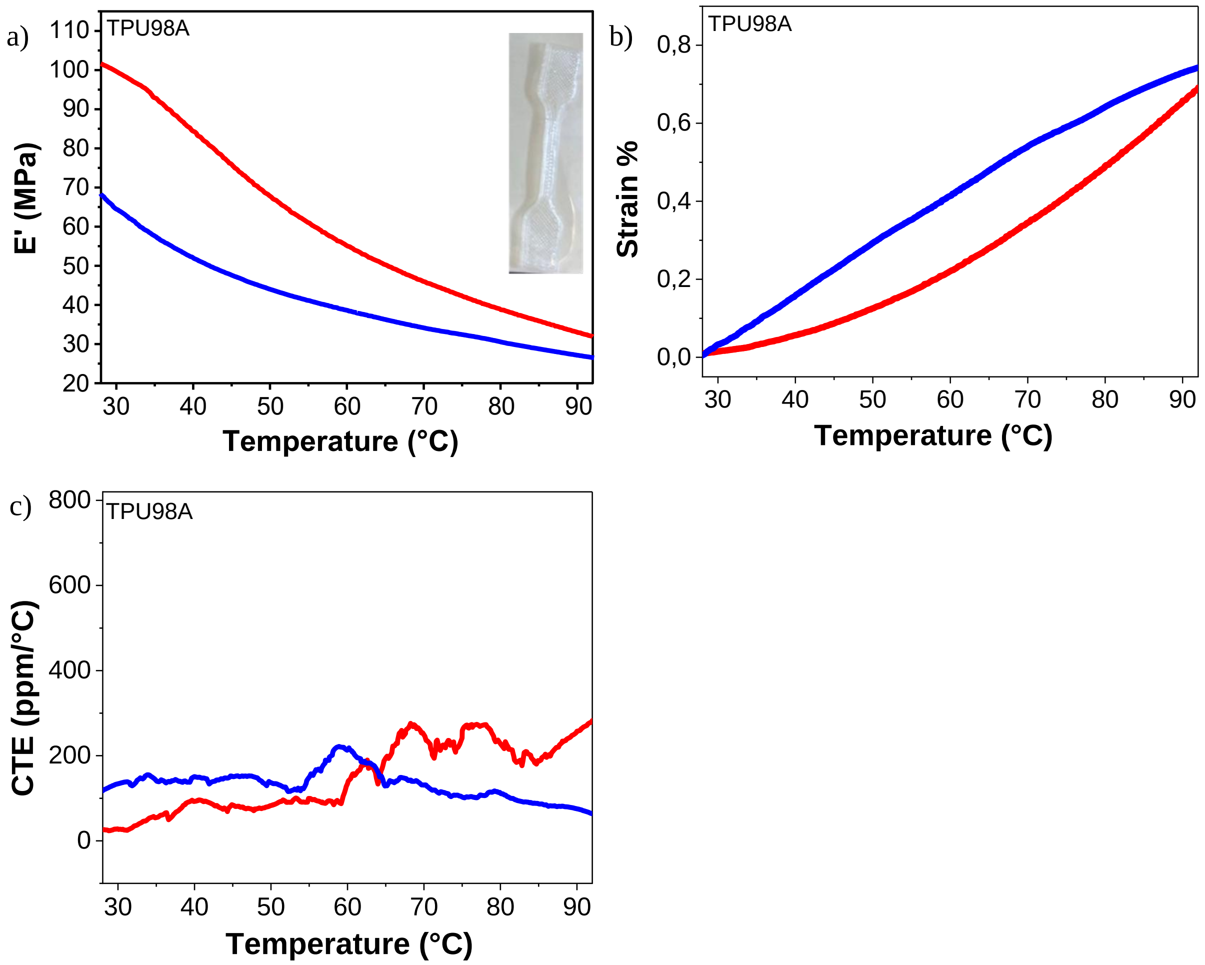

Section S3. Theoretical analysis of the metastructures

Definition of CTE for metastructures

CTE denotes a material's geometric dependence on temperature under specified thermodynamic conditions. Most materials will expand when they undergo a positive temperature change. Mathematically, the CTE tensor, α_{ij}, is the temperature derivative of the Cauchy strain tensor, ε_{ij}, under constant pressure:

$$\alpha_{ij} = \left(\frac{\partial \varepsilon_{ij}}{\partial T}\right)_p \quad (1)$$

By assuming a linear dependence of strain on temperature (which is accurate for small temperature changes, around non-cryogenic temperatures) we can write:

$$\alpha_{ij} = \frac{\varepsilon_{ij}}{\Delta T} \quad (2)$$

Ignoring shear strains, we can compute the CTE along a particular direction using the following expression:

$$\alpha = \frac{l_f - l_i}{l_i(T_f - T_i)} \quad (3)$$

Using Equation (3), in which *l* denotes length along a specific direction and subscripts f and i denote the final and initial configurations respectively, one can compute the CTE of any material along that direction by applying a temperature change and measuring the original length and change in length of an infinitesimally thin segment along that direction. This definition of CTE is applicable and useful when defining thermal expansion between atoms in a crystal. In this way, we use this definition of CTE to characterize the thermal response of metastructures. In the same way that atomic unit cells are building blocks for crystals, structural unit cells are building blocks for metastructures. The CTE of metastructures is thus defined discreetly, between nodes of unit cells within the metastructure.

Analytical calculation of the CTE of the bi-material diamond structures

For the theoretical analysis model, the assumptions and simplifications are given as follows:

(1) The RVE (Representative Volume Element) is an element under the periodic array structure and does not need to consider the boundary effect.
(2) Deformation of the structure includes bending, tensile, and shear modes.
(3) Deformation of the structure is in the elastic range.
(4) The base members and hypotenuse members are rigid connections.

Due to the symmetry of the structure, the triangular lattice is selected for theoretical calculations. Within the range of elastic deformation, as shown in **Scheme S3c** and **S3d**, the deformation of the RVE consists of two steps of thermal expansion and internal stress F when submitted to a uniform temperature change ΔT. The horizontal displacement ΔL_{Tx} and vertical displacement ΔL_{Ty} of point A and point B with respect to the original position are caused by

thermal expansion first, shown in **Scheme S3b**. Then, point A is moved horizontally by ΔL_{Fx} and point B vertically by ΔL_{Fy} under the action of F and M, as shown in **Scheme S3d**. The displacements of point A in the X- and Y-directions are represented by u_x^A and u_y^A, respectively, and the rotation angle is represented by θ_A. The overlap effects between the base member and the hypotenuse member at the joints can be ignored when the bar is slender, while it must be considered when the bar is short and thick. Therefore, the effective lengths of the base member and the hypotenuse member are defined as L_1^* and L_2^*, as illustrated in Eqs. (4) and (5) below:

$$L_2^* = L_2 - \left(\frac{t_2}{sin(\theta)}\right) \tag{4}$$

$$L_1^* = 2L_2cos(\theta) - \frac{2t_1}{tan\theta} \tag{5}$$

Scheme S3. Deformation of the metastructure by changing temperature: (a) array of diamond unit cells, (b) RVE (represented by ABC) along with its geometrical parameters, (c) deformation caused by thermal expansion, (d) deformation caused by mechanical forces, (e) boundary conditions and force method of the bar deformation.

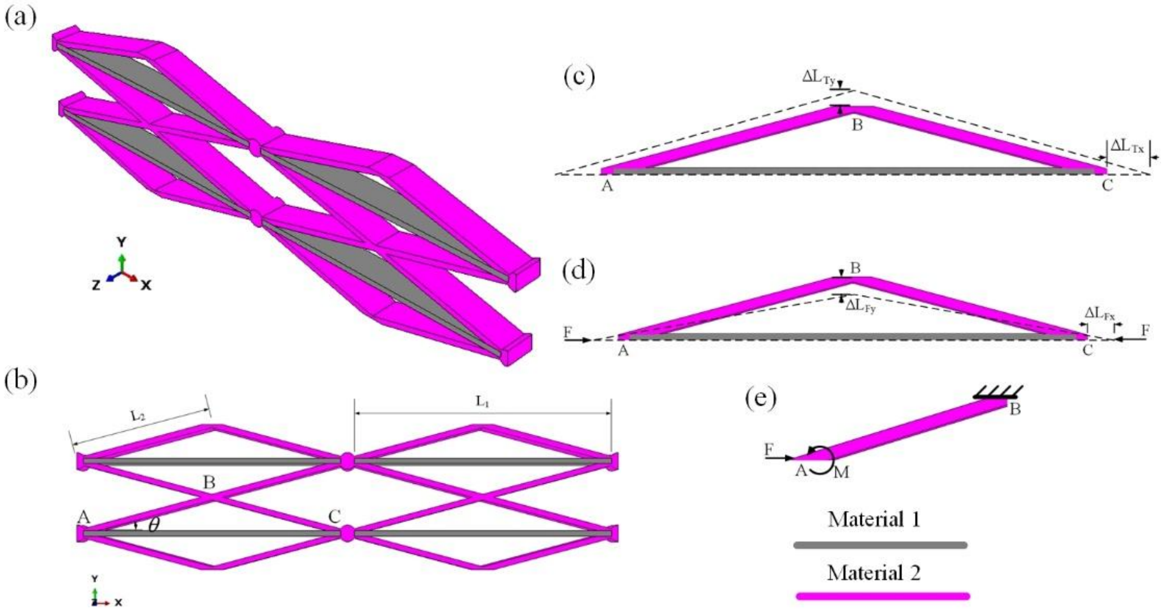

According to the geometric features and the deformation coordination condition, the displacements of u_x^A and u_y^A can be expressed as

$$u_x^A = \Delta L_{Tx} - 2\Delta L_{Fx} = \frac{FL_1^*}{E_1A_1} \tag{6}$$

$$u_y^A = \Delta L_{Ty} - \Delta L_{Fy} \tag{7}$$

The CTE in the Y direction can be expressed as

$$\alpha_y = \frac{u_y^A}{\Delta T L_2^* sin\theta} = \alpha_2 - \frac{\sigma_1\sigma_2}{\Delta T L_2^* sin\theta} \tag{8}$$

where σ_1 and σ_2 are defined as follows:

$$\sigma_1 = \frac{E_1A_1(\alpha_1 - \alpha_2)\Delta T}{1 + \frac{2E_1A_1\sigma_2}{L_1^*}}$$

$$\sigma_2 = \frac{{L_2^*}^3 sin^2\theta}{36E_2I_2} + \frac{L_2^* cos^2\theta}{3E_2A_2} + \frac{kL_2^* sin^2\theta}{3G_2A_2}$$

In these equations ΔT is the temperature change, E_i, G_i, and ν_i are the Young's modulus, shear modulus, and Poisson's ratio of the member material i, respectively. A_i is the cross-section area, I_i is the second moment of area, and k is the shape factor of the cross-section, which is equal to 1.2 for rectangular cross-section.

$$G_i = \frac{E_i}{2(1+\nu_i)}$$
$$A_i = t_i b_i$$
$$I_i = \frac{b_i t_i^3}{12}$$

By substituting the above variables into Eq.(8), we end up with the following equations:

$$M_f = \frac{\alpha_y}{\alpha_2} = 1 - \frac{\frac{E_1 A_1}{E_2 A_2}\left(\frac{\alpha_1}{\alpha_2} - 1\right)\cos^2\theta\left(1+2k(1+\nu_2)\tan^2\theta + \frac{{L_2^*}^2\tan^2\theta}{t_2^2}\right)}{\left(3 + \frac{2E_1 A_1 L_2^*}{E_2 A_2 L_1^*}\cos^2\theta\left(1+2k(1+\nu_2)\tan^2\theta + \frac{{L_2^*}^2\tan^2\theta}{t_2^2}\right)\right)sin(\theta)} \quad (9)$$

$$M_f^N = \frac{\alpha_y}{\alpha_1} = 1 - \frac{\frac{E_2 A_2}{E_1 A_1}\left(\frac{\alpha_2}{\alpha_1} - 1\right)\cos^2\theta\left(1+2k(1+\nu_1)\tan^2\theta + \frac{{L_1^*}^2\tan^2\theta}{t_1^2}\right)}{\left(3 + \frac{2E_2 A_2 L_1^*}{E_1 A_1 L_2^*}\cos^2\theta\left(1+2k(1+\nu_1)\tan^2\theta + \frac{{L_1^*}^2\tan^2\theta}{t_1^2}\right)\right)sin(\theta)} \quad (10)$$

In the above equations M_f is the magnification factor of the diamond unit cell when the outer struts are made from material 2, which has higher CTE, and M_f^N is for the case that the outer struts are made of material 1 which has lower CTE. (Note that we use a different definition for 1 and 2 in the manuscript.)

Table S3. Input parameters for the analytical simulations. Note: The values of A_1 and A_2 are set to 1 for simplicity because only their ratio affects the simulation outcome.

Variable	Description	Value
α_1	CTE1 (ppm/°C)	130
α_2	CTE2 (ppm/°C)	640
E_1	Young's modulus 1 (MPa)	45
E_2	Young's modulus 2 (MPa)	12
A_1	Cross-section area 1	1
A_2	Cross-section area 2	1
θ	Internal angle (degrees)	15
k	Shape factor	1.2
$\nu_1 = \nu_2$	Poisson coefficient	0.3 (estimated)
t_1	Bar thickness 1 (mm)	0.6
t_2	Bar thickness 2 (mm)	0.6
L_2	Length 2 (mm)	12
L_1^*	Effective length 1 (mm)	19.45
L_2^*	Effective length 2 (mm)	10.26
ΔT	Temperature change (°C)	35

Finite Element Analysis

We conducted a Finite Element Method (FEM) analysis of our structures using full three-dimensional (3D) models to accurately represent the geometry of the printed structures, including their thickness (~0.8 mm) with COMSOL Multiphysics, which offers an integrated platform for modelling coupled physical processes. This approach avoids the simplifying assumptions associated with two-dimensional plane stress or plane strain formulations and enables a more realistic prediction of the thermomechanical response. Our strategy was motivated by the analogy between the spontaneous strain associated with the spin-crossover and the ordinary thermal expansion phenomenon. This analogy allows established approaches, developed for architected materials to modulate the coefficient of thermal expansion, to be directly adapted for SCO-based systems.

In our approach, two distinct materials were integrated into the metastructure: an SCO composite showing a high thermal-strain response, and a second, inactive material characterized by a comparatively small thermal strain. Embedding these materials into a carefully engineered lattice permits the effective strain of the structure to be tuned and, in certain configurations, amplified. This bi-material strategy enables the development of SCO-based structures with programmable thermal strain, covering a broad operational spectrum. We used multiphysics coupling to predict the thermal expansion behaviour. We coupled the solid mechanics interface with the heat transfer in solids interface to model the thermal expansion.

Solid mechanics is formulated on the general principles of continuum mechanics and involves the solution of the equations of motion in combination with a constitutive description of the solid. The heat transfer in solids interface accounts for conduction, convection, and radiation within the material domain. By coupling these two physics interfaces, the thermal expansion response of the structure was successfully modelled. We initiated the simulations by constructing the geometry of the metastructures. We considered two configurations: in the first, three columns are connected in parallel, each comprising seven diamond-shaped unit cells; in the second, a single column was modelled, also containing seven diamond unit cells. We selected the geometries to investigate the role of structural dimensionality in amplifying and modulating the effective strain response. For the material properties, we assigned two distinct classes within the simulations. The first was the active material, corresponding to the SCO compound, which was modelled through the thermal-strain analogy to reproduce its unusually high spontaneous strain. The second was a passive material, which exhibited a comparatively negligible strain. For this purpose, we used Thermoplastic Polyurethane (TPU), whose properties are listed in **Table S4** alongside those of the SCO material, which are primarily obtained from experimental measurements, supplemented by commonly reported values from the literature. (N.B. The common parameters with the analytical model (cf. **Table S3**) were kept identical.)

A linear elastic material model was employed for both the TPU and the SCO material and the reported strains correspond to engineering strains. Although the overall deformation of the metastructures reaches values of up to ~7%, the corresponding local strains within the material remain below ~3%, which is within the typical validity range of linear elasticity. This distinction ensures the appropriateness of the chosen material model. Therefore, the difference between engineering strain and true strain is negligible in this context, and the chosen strain measure does not significantly affect the results.

Additionally, the assumption of linear elastic behavior is supported by the good reversibility of the thermal expansion and contraction observed experimentally, indicating negligible plasticity or material nonlinearity.

In the initial configuration, we assigned the active material to the diamond-shaped struts, while the passive TPU occupied the middle struts. In the inverse configuration, the assignment was reversed: the active SCO material was placed in the middle struts, whereas the passive TPU formed the diamond struts. This contrast between the two arrangements allowed us to evaluate the role of material distribution in determining the effective thermal strain of the metastructure. Boundary conditions were then applied by rigidly clamping the bottom of the structure, thereby preventing any displacement or rotation at this boundary and simulating a fixed support. The rest of structure was left unconstrained, allowing free deformation in response to thermal expansion. Meshing was performed using a tetrahedral discretization, which enabled the accurate resolution of complex geometrical features and provided reliable convergence during the simulations. The finite element mesh was generated using COMSOL's physics-controlled meshing with a "normal" element size. This results in an unstructured mesh that is automatically adapted to the geometry and governing physics. Such an approach provides a good balance between computational efficiency and accuracy for complex three-dimensional lattice structures. A mesh sensitivity analysis confirmed that further refinement did not significantly influence the simulation results. The results demonstrated distinct strain responses depending on the structural configuration and material distribution (**Figure S10**).

For the three-column structure with active diamond struts and inactive middle struts, the structure showed a strain of 22 % with a magnification factor of 10. Conversely, when the material distribution was inverted i.e. placing the inactive material in the diamond struts and the active material in the middle struts, the structure contracted by 17 %, corresponding to a magnification factor of -7.7. A similar trend was observed in the single-column structure. With active diamond struts and inactive middle struts, the structure showed a strain of 22 % corresponding to a magnification factor of 10. When the material distribution was again inverted i.e. placing the inactive material in the diamond struts and the active material in the middle struts, the single-column structure contracted by 18 %, corresponding to a magnification factor of -8.3. These findings highlight the sensitivity of the effective thermal strain to both structural dimensionality and material placement, underscoring the versatility of the proposed bi-material metastructures. In the same time, no substantial differences in the behaviour of the 1D and 2D lattices have been noted.

Table S4. FEM simulation parameters

	Property	Value
**Active SCO Material**	Density	1.448 kg/m³
	Heat capacity at constant pressure	1800 J/(kg·K)
	Thermal conductivity	0.2 W/(m·K)
	Young's modulus	12E6 Pa
	Poisson's ratio	0.3
	Thermal strain	0.022
	Coefficient of thermal expansion	640E-6 1/K

	Property	Value
**Inactive TPU98A**	Density	1.2 kg/m³
	Heat capacity at constant pressure	1800 J/(kg·K)
	Thermal conductivity	0.2 W/(m·K)
	Young's modulus	45E6 Pa
	Poisson's ratio	0.3
	Thermal strain	0.00455
	Coefficient of thermal expansion	130E-6 1/K

Figure S10. FEA simulation results for the single column and multi-column lattices, including both the positive and negative CTE designs.

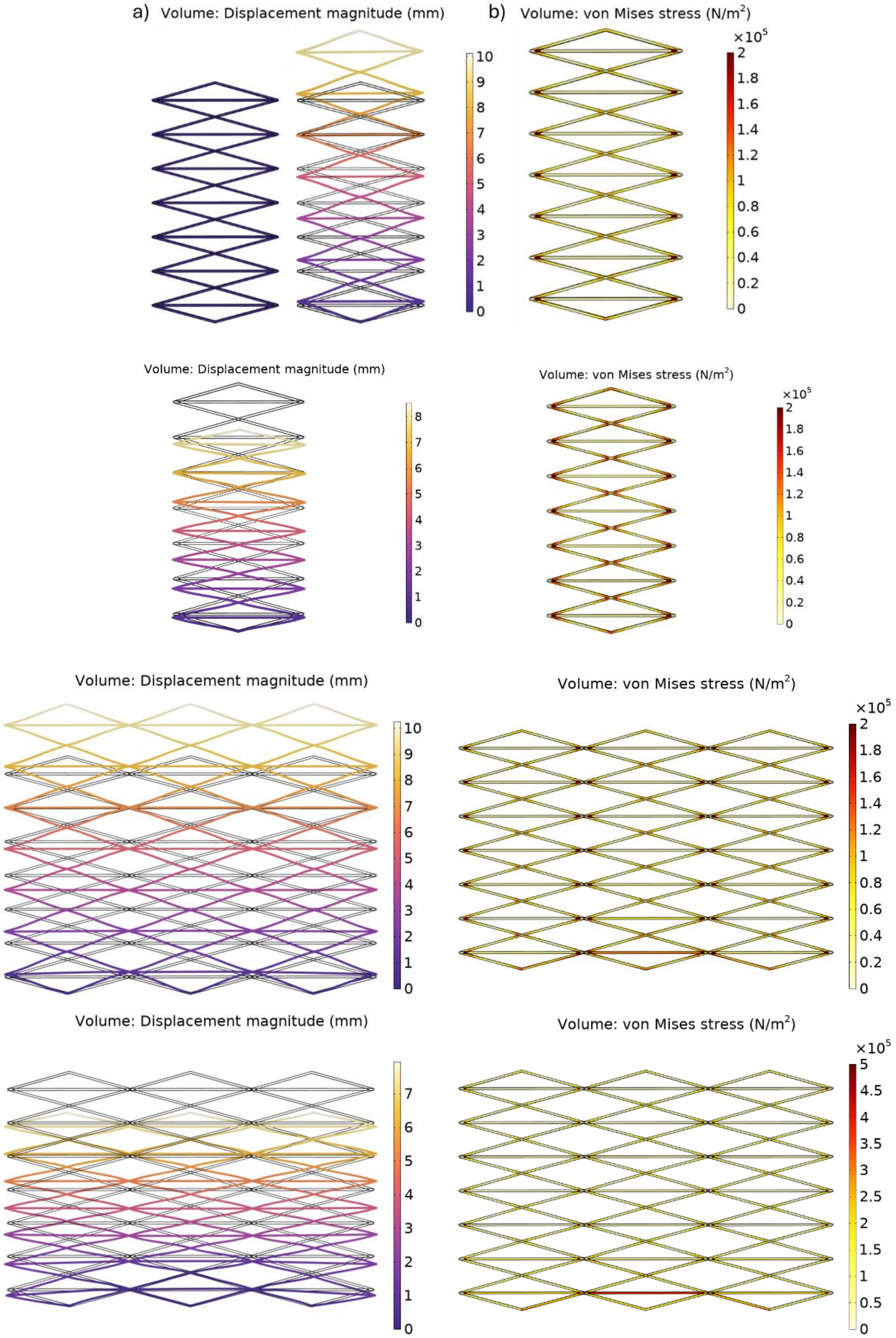

Section S4. Experimental characterization of the metastructures

Figure S11. Photographs of a positive CTE bi-material metastructure (white: TPU98A, pink/yellow: 1@TPU70A) recorded at (a) 25 °C, (b) 60 °C and (c) 95 °C.

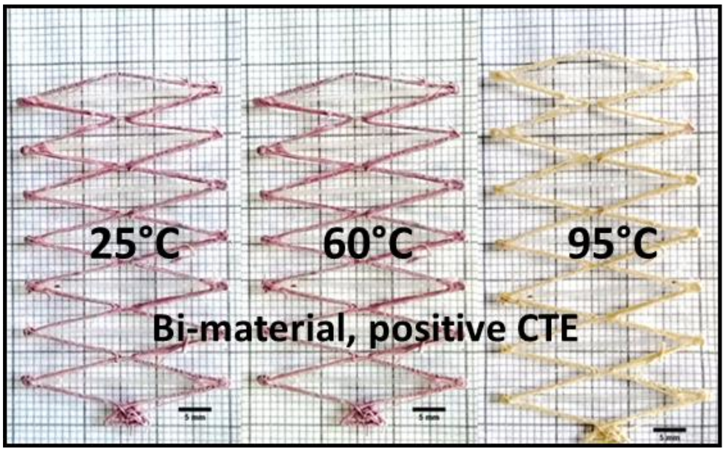

Figure S12. Programmable positive and negative thermal actuation in architected spin crossover structures (1D lattice). (a, e) Diamond structure made of **1**@TPU70A (pink) without inner struts, (b, f) Bi-material structure with **1**@TPU70A outer struts and TPU98A (transparent) inner struts, (c, g) Inverse structure with TPU98A outer struts and **1**@TPU70A inner struts, (d, h) Bi-material structure with **1**@TPU70A outer struts and polylactic acid (PLA, black) inner struts. The photos in the top and bottom rows were acquired at 25 and 95 °C, respectively. The color change of the members from pink to yellowish denotes the spin transition from the LS to the HS state.

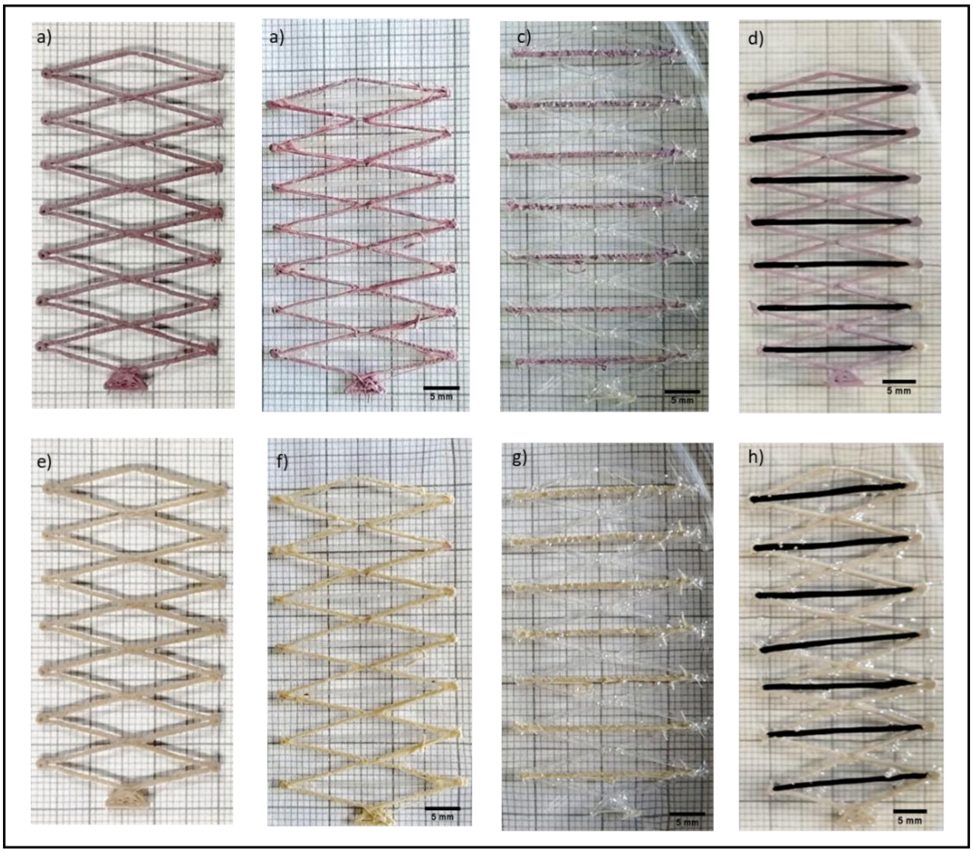

Figure S13. Programmable positive and negative thermal actuation in architected spin crossover structures (1D lattice) for three successive thermal cycles. (a-g) Diamond structure made of **1**@TPU70A (pink) without inner struts, (h-n) Bi-material structure with **1**@TPU70A (pink) outer struts and TPU98A (transparent) inner struts, (o-u) Inverse structure with TPU98A outer struts (transparent) and **1**@TPU70A (pink) inner struts. The color change of the members from pink to yellowish denotes the spin transition from the LS to the HS state.

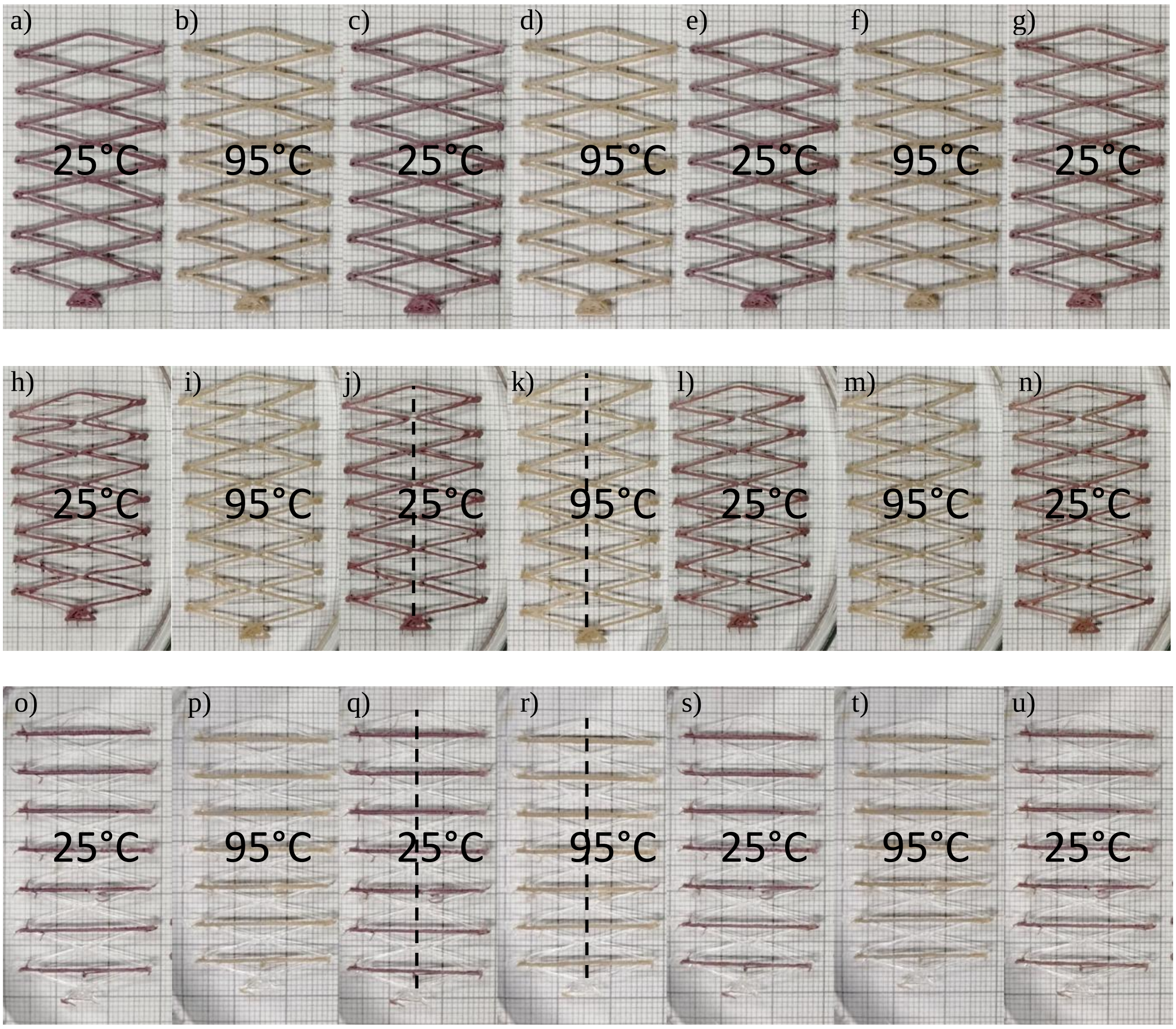

Supplement: Supplementary file 1 — Supporting File: adma73061‐sup‐0001‐SuppMat.pdf. [file ADMA-38-e22073-s001.pdf]
